# Supplementary material for: Mutation Load in Sunflower Inversions Is Negatively Correlated with Inversion Heterozygosity
Source: Mol Biol Evol. 2022 May 10;39(5):msac101. doi: 10.1093/molbev/msac101 (PMC9127631; doi:10.1093/molbev/msac101)
Supplement: msac101_Supplementary_Data [file msac101_supplementary_data.pdf]

Table S1. Sample information for the newly-sequenced individuals in this study.

Population	Sample ID	Ecotype	Coordinate
GSD	GSD983	non-dune	37.663 N 105.625 W
	GSD1009	dune	37.774 N 105.576 W
	GSD1039	dune	37.787 N 105.569 W
	GSD1085	non-dune	37.774 N 105.598 W
	GSD1139	dune	37.818 N 105.597 W
	GSD1169	non-dune	37.836 N 105.603 W
	GSD1184	dune	37.773 N 105.553 W
	GSD1258	dune	37.786 N 105.531 W
	GSD1276	dune	37.764 N 105.524 W
	GSD1329	dune	37.745 N 105.546 W
	GSD1375	non-dune	37.716 N 105.533 W
	GSD1503	non-dune	37.765 N 105.615 W
	GSD1573	dune	37.761 N 105.57 W
	GSD1707	dune	37.803 N 105.524 W
	GSD1732	dune	37.807 N 105.554 W
	GSD1795	non-dune	37.813 N 105.515 W
	GSD2008	non-dune	37.757 N 105.507 W
	GSD2031	non-dune	37.758 N 105.509 W
	GSD2067	dune	37.758 N 105.515 W
	GSD2252	non-dune	37.672 N 105.592 W
MON	MON014	dune	31.63151 N 102.80999 W
	MON069	dune	31.63679 N 102.81361 W
	MON106	dune	31.64052 N 102.81797 W
	MON151	dune	31.64075 N 102.81345 W
	MON220	dune	31.62984 N 102.81567 W
	MON263	non-dune	31.61642 N 102.81197 W
	MON308	non-dune	32.3204 N 103.82318 W
	MON365	non-dune	32.21017 N 103.58173 W
	MON401	non-dune	32.17832 N 103.38889 W
	MON459	non-dune	32.0889 N 103.17894 W
	MON520	dune	32.03659 N 103.15085 W
	MON564	dune	32.03527 N 103.15001 W
	MON606	non-dune	31.99352 N 103.13029 W
	MON658	non-dune	31.82584 N 103.07813 W
	MON713	non-dune	31.64043 N 102.98539 W
	MON768	non-dune	31.58296 N 102.87074 W
	MON818	non-dune	31.61138 N 102.83128 W
	MON862	dune	31.62488 N 102.81174 W

GSD: Great Sand Dunes National Park and Preserve; MON: Monahans Sandhills State Park.

Table S2. Location, sample size and genotype frequencies of each inversion of each population in *Helianthus annuus*. The genotype frequencies are presented as (0/0)/(0/1)/(1/1).

Population	Latitude	Longitude	Sample #	ann01.01	ann05.01	ann13.01	ann15.01
ANN_01	38.56535	-121.63765	10	0.5/0.5/0	0.4/0.6/0	0.1/0.4/0.5	0.6/0.3/0.1
ANN_02	36.59499	-120.75822	10	1/0/0	0.8/0.1/0.1	0/0.4/0.6	0.4/0.6/0
ANN_03	36.24542	-120.31654	10	1/0/0	0.6/0.4/0	0.6/0.2/0.2	0.2/0.8/0
ANN_04	34.75087	-119.07235	10	1/0/0	1/0/0	0.6/0.4/0	0.2/0.8/0
ANN_05	33.69278	-116.18411	10	1/0/0	0.7/0.3/0	0.4/0.4/0.2	0.9/0.1/0
ANN_06	34.24976	-117.45148	10	1/0/0	1/0/0	0.2/0.6/0.2	0.3/0.5/0.2
ANN_07	38.7892	-119.9363	10	0.7/0.3/0	0.5/0.5/0	1/0/0	0.6/0.3/0.1
ANN_08	38.67722	-119.73895	10	0.8/0.2/0	0.4/0.6/0	0.9/0.1/0	0.6/0.3/0.1
ANN_09	37.43321	-118.40646	10	1/0/0	0.1/0.4/0.5	0.1/0.4/0.5	0.4/0.5/0.1
ANN_10	37.17346	-118.27904	10	0.9/0.1/0	0.4/0.4/0.2	0/0.4/0.6	0.9/0.1/0
ANN_11	35.19566	-114.06666	10	1/0/0	0.1/0.6/0.3	0.5/0.4/0.1	0.1/0.5/0.4
ANN_12	35.2533	-112.13355	10	1/0/0	0.3/0.5/0.2	0.8/0.2/0	0.2/0.7/0.1
ANN_13	34.75056	-112.01697	10	1/0/0	0.8/0.2/0	0.1/0.5/0.4	0.1/0.6/0.3
ANN_14	36.1086	-111.24184	10	1/0/0	0.6/0.4/0	0/0.3/0.7	0.9/0.1/0
ANN_15	35.03852	-110.7351	10	1/0/0	0/0.3/0.7	0.2/0.7/0.1	0.1/0.6/0.3
ANN_16	35.40041	-108.23557	10	1/0/0	0.2/0.3/0.5	0.2/0.4/0.4	0.6/0.3/0.1
ANN_17	35.24503	-107.68594	10	1/0/0	0.7/0.3/0	0.5/0.2/0.3	0.3/0.6/0.1
ANN_18	36.07308	-104.28873	10	0.9/0.1/0	0.2/0.5/0.3	0.5/0.5/0	0.2/0.5/0.3
ANN_19	36.7268	-104.01913	10	1/0/0	0.2/0.6/0.2	0.2/0.4/0.4	0.5/0.4/0.1
ANN_20	38.44366	-107.62928	10	1/0/0	0.3/0.1/0.6	0.3/0.2/0.5	0.8/0.2/0
ANN_21	38.91004	-108.3738	10	1/0/0	0.1/0.4/0.5	0/0.1/0.9	0.8/0.2/0
ANN_22	39.00672	-108.46384	10	0.9/0.1/0	0.5/0.5/0	0.1/0.4/0.5	0.5/0.4/0.1
ANN_23	40.66257	-116.23876	10	0.7/0.3/0	0.9/0.1/0	0.5/0.3/0.2	0.4/0.3/0.3
ANN_24	39.48892	-119.99511	10	0.4/0.3/0.3	0.6/0.4/0	0.5/0.5/0	0.5/0.5/0
ANN_25	36.79654	-114.09841	10	1/0/0	0.2/0.4/0.4	0.1/0.4/0.5	0.7/0.3/0
ANN_26	37.13733	-113.51348	10	1/0/0	0/0.7/0.3	0.8/0.2/0	0.4/0.2/0.4
ANN_27	37.13137	-112.56561	10	1/0/0	0.4/0.4/0.2	0/0.1/0.9	1/0/0
ANN_28	38.21652	-112.64767	10	1/0/0	0.5/0.5/0	0.2/0.4/0.4	0.6/0.4/0
ANN_29	39.55006	-112.35661	10	1/0/0	0.9/0.1/0	0/0/1	0.7/0.3/0
ANN_30	39.7148	-112.20662	10	1/0/0	0.9/0.1/0	0.4/0.5/0.1	0.1/0.3/0.6
ANN_31	40.1781	-111.65071	10	1/0/0	1/0/0	0.3/0.5/0.2	0.5/0.3/0.2
ANN_32	41.48524	-112.0556	10	1/0/0	0.8/0.1/0.1	0/0.1/0.9	0.9/0.1/0
ANN_33	40.77115	-112.15004	10	0.9/0.1/0	1/0/0	0.3/0.3/0.4	0.6/0.4/0
ANN_34	40.69149	-112.26423	10	1/0/0	0.7/0.3/0	0/0.1/0.9	0.6/0.3/0.1
ANN_35	40.51274	-109.51947	10	1/0/0	0.5/0.4/0.1	0.1/0.3/0.6	0.3/0.4/0.3
ANN_36	41.77426	-107.45885	10	1/0/0	0.6/0.3/0.1	0.2/0.4/0.4	0.3/0.6/0.1
ANN_37	40.48679	-105.09619	10	1/0/0	0/0.8/0.2	0/0/1	0.5/0.5/0
ANN_38	38.44118	-98.63231	10	0.5/0.4/0.1	0/0.2/0.8	0.1/0.4/0.5	0.8/0.2/0
ANN_39	37.45911	-97.9179	10	0.3/0.6/0.1	0/0.9/0.1	0.2/0.2/0.6	0.8/0.2/0
ANN_40	37.23327	-98.66459	10	0.2/0.4/0.4	0.6/0.4/0	0.5/0.3/0.2	0/0.5/0.5
ANN_41	36.80874	-98.22242	10	0.3/0.5/0.2	0.1/0.4/0.5	0/0.3/0.7	0.3/0.6/0.1
ANN_42	32.92224	-97.36547	10	0.2/0.5/0.3	0.3/0.3/0.4	0.8/0.2/0	0/0.6/0.4
ANN_43	29.73315	-97.65574	10	0/0.2/0.8	0.1/0.4/0.5	0.8/0.2/0	0/0/1

Population	Latitude	Longitude	Sample #	ann01.01	ann05.01	ann13.01	ann15.01
ANN_44	29.05186	-99.01808	10	0/0.2/0.8	0.4/0.4/0.2	1/0/0	0/0/1
ANN_45	28.63085	-100.1189	10	0/0/1	0/0.1/0.9	0.8/0.2/0	0/0/1
ANN_46	33.92321	-116.86432	10	0.9/0.1/0	1/0/0	0.1/0.7/0.2	0.8/0.2/0
ANN_47	32.73158	-111.53769	10	0.7/0.3/0	0.5/0.5/0	0.2/0.5/0.3	0.3/0.7/0
ANN_48	31.5081	-111.01274	10	0.6/0.4/0	0.8/0.2/0	0.2/0/0.8	0.3/0.2/0.5
ANN_49	31.43996	-110.10544	10	1/0/0	0.7/0.3/0	0.3/0.5/0.2	0.7/0.3/0
ANN_50	32.17943	-108.38541	10	1/0/0	0.9/0.1/0	0/0.5/0.5	0.8/0.2/0
ANN_51	32.12876	-106.67373	10	1/0/0	0.2/0.6/0.2	0/0/1	1/0/0
ANN_52	31.13312	-104.02012	10	0.1/0.7/0.2	0.5/0.3/0.2	0.5/0.4/0.1	0/0.3/0.7
ANN_53	31.03955	-103.13683	10	0.1/0.9/0	0.3/0.5/0.2	0.4/0.6/0	0.1/0.2/0.7
ANN_54	29.11355	-97.39837	10	0.1/0.4/0.5	0/0.1/0.9	1/0/0	0/0/1
ANN_55	28.66061	-96.58348	10	0/0.3/0.7	0/0.4/0.6	1/0/0	0/0/1
ANN_56	28.08852	-97.06011	10	0/0/1	0.6/0.4/0	0.9/0.1/0	0/0/1
ANN_57	27.63854	-97.24526	10	0.1/0.4/0.5	0.1/0.6/0.3	1/0/0	0/0/1
ANN_58	28.24166	-97.67443	10	0/0.1/0.9	0.3/0.4/0.3	1/0/0	0/0/1
ANN_59	26.50251	-97.77181	10	0.2/0.6/0.2	0/0/1	1/0/0	0/0/1
ANN_60	27.28522	-97.66729	10	0/0.3/0.7	0/0.7/0.3	1/0/0	0/0/1
ANN_61	27.77652	-97.66801	9	0.11/0.67/0.22	0.11/0.56/0.33	1/0/0	0/0/1
ANN_62	42.24842	-96.05867	12	0.25/0.75/0	0/0.17/0.83	0/0.08/0.92	1/0/0
ANN_63	41.04647	-95.82286	11	0.45/0.45/0.1	0/0.27/0.73	0.08/0.17/0.75	1/0/0
ANN_64	39.68894	-99.06605	11	0.64/0.36/0	0/0.33/0.67	0/0.25/0.75	0.83/0.17/0
ANN_65	39.82806	-99.9072	16	0.75/0.125/0.125	0.125/0.3125/0.5625	0.0625/0.125/0.8125	0.6875/0.25/0.0625
ANN_66	49.29649	-100.40875	10	1/0/0	0.2/0.4/0.4	0/0/1	1/0/0
ANN_67	40.07698	-94.86874	10	0.6/0.4/0	0.1/0.1/0.8	0/0.18/0.82	0.73/0.27/0
ANN_68	47.24255	-101.04321	10	0.9/0.1/0	0.1/0.7/0.2	0.3/0.1/0.6	1/0/0
ANN_69	44.50105	-100.0715	9	0.67/0.22/0.11	0/0.44/0.56	0/0.11/0.89	1/0/0
ANN_70	42.8228	-96.78587	12	0.67/0.33/0	0.17/0.33/0.5	0/0.17/0.83	1/0/0
ANN_71	49.23184	-102.9884	12	0.92/0/0.08	0/0.58/0.42	0.08/0/0.92	1/0/0

Table S3. Location, sample size and genotype frequencies of each inversion of each population in *Helianthus argophyllus*. The genotype frequencies are presented as (0/0)/(0/1)/(1/1).

Population	Latitude	Longitude	Sample #	arg10.01
ARG_01	29.6465	-97.69094	10	1/0/0
ARG_02	29.43897	-97.73836	10	1/0/0
ARG_03	29.31939	-97.7441	10	1/0/0
ARG_04	29.60147	-97.70881	10	1/0/0
ARG_05	29.45132	-97.6954	10	1/0/0
ARG_06	29.27191	-97.66967	10	1/0/0
ARG_07	28.70013	-97.38772	10	1/0/0
ARG_08	28.73672	-97.03155	10	1/0/0
ARG_09	28.18143	-96.9948	10	1/0/0
ARG_10	28.10813	-97.02884	10	0.4/0.5/0.1
ARG_11	27.99705	-97.07636	10	0.4/0.4/0.2
ARG_12	27.93269	-97.12295	10	0.1/0.3/0.6
ARG_13	27.83272	-97.04939	10	0/0.9/0.1
ARG_14	27.8469	-97.04873	10	0.4/0.4/0.2
ARG_15	27.83481	-97.08834	10	0.7/0.2/0.1
ARG_16	27.68602	-97.17672	10	0/0.3/0.7
ARG_17	27.61823	-97.21178	9	0.78/0.22/0
ARG_18	27.47822	-97.28085	10	0.1/0.1/0.8
ARG_19	27.57942	-97.2215	10	0/0.3/0.7
ARG_20	27.6611	-97.31138	10	0.4/0.3/0.3
ARG_21	28.12154	-97.39245	10	1/0/0
ARG_22	28.27849	-97.29757	10	1/0/0
ARG_23	26.8902	-98.13698	10	1/0/0
ARG_24	26.92402	-97.79218	10	1/0/0
ARG_25	27.15959	-97.79411	10	1/0/0
ARG_26	27.27342	-97.80311	10	1/0/0
ARG_27	27.14245	-98.14997	10	1/0/0
ARG_28	27.00215	-98.13579	10	1/0/0
ARG_29	27.10014	-98.14704	10	1/0/0
ARG_30	27.25741	-97.94589	10	1/0/0

Table S4. Location, sample size and genotype frequencies of each inversion of each population in *Helianthus petiolaris*. The genotype frequencies are presented as (0/0)/(0/1)/(1/1).

Population	Latitude	Longitude	Sample #	pet05.01	pet09.01	pet11.01	pet14.01
GSD	37.773	-105.553	20	0.65/0.2/0.15	0.3/0.3/0.4	0.65/0.1/0.25	0/0.25/0.75
Mon	31.61642	-102.81197	18	0/0.17/0.83	0.22/0.33/0.44	0.61/0.22/0.17	0.44/0.22/0.33
PET_01	35.1371	-111.64211	10	0/0/1	1/0/0	0/0/1	0/0/1
PET_02	34.91703	-111.90977	10	0/0/1	1/0/0	0/0/1	0/0/1
PET_03	35.33119	-111.54146	10	0/0/1	1/0/0	0/0/1	0/0/1
PET_04	35.39031	-111.57754	10	0/0/1	1/0/0	0/0/1	0/0/1
PET_05	35.15387	-109.50207	10	0/0/1	1/0/0	0/0/1	0/0/1
PET_06	35.51651	-105.68484	10	0/0/1	1/0/0	0/0/1	0/0/1
PET_07	36.49673	-104.57014	10	0/0/1	1/0/0	0/0/1	0/0/1
PET_08	37.52901	-105.297	10	0/0/1	1/0/0	0/0/1	0/0/1
PET_09	37.66278	-105.64088	8	0/0.125/0.875	0.875/0.125/0	0.125/0.5/0.375	0/0.125/0.875
PET_10	38.47747	-106.08848	10	0/0/1	1/0/0	0/0/1	0/0/1
PET_11	38.48952	-107.03795	9	0/0/1	1/0/0	0/0/1	0/0/1
PET_12	36.90004	-112.91885	9	0/0/1	1/0/0	0/0/1	0/0/1
PET_13	37.23794	-112.85064	9	0/0/1	1/0/0	0/0/1	0/0/1
PET_14	40.55035	-109.91641	10	0/0/1	1/0/0	0/0/1	0/0/1
PET_15	40.69709	-105.61168	10	0/0/1	1/0/0	0/0/1	0/0/1
PET_16	40.38047	-104.52282	10	0.1/0.1/0.8	0.8/0.2/0	0.4/0.4/0.2	0/0/1
PET_17	40.15158	-104.60307	10	0.1/0.3/0.6	0.5/0.3/0.2	0.1/0.7/0.2	0/0/1
PET_18	39.9137	-104.61456	10	0/0.4/0.6	0.2/0.5/0.3	0.4/0.5/0.1	0/0/1
PET_19	39.63705	-104.33484	10	0/0.1/0.9	0.6/0.3/0.1	0.7/0.2/0.1	0/0/1
PET_20	38.3336	-98.83812	10	0/0.6/0.4	0.7/0.3/0	0.1/0.3/0.6	0/0/1
PET_21	38.33349	-98.69159	10	0.1/0.5/0.4	0.2/0.5/0.3	0.8/0.2/0	0/0/1
PET_22	38.56505	-97.96174	10	0.1/0.3/0.6	0.4/0.5/0.1	0.7/0.3/0	0/0/1
PET_23	37.676	-97.40646	9	0.44/0.33/0.22	0.22/0.67/0.11	0.33/0.56/0.11	0/0/1
PET_24	37.37939	-97.8877	6	0.33/0.33/0.33	0.67/0.33/0	0.17/0.5/0.33	0/0/1
PET_25	36.83371	-98.6481	10	0.1/0.6/0.3	0.6/0.3/0.1	0.2/0.7/0.1	0/0/1
PET_26	36.56341	-98.88941	9	0.78/0.11/0.11	0.11/0.44/0.44	0.78/0.22/0	0/0.22/0.78
PET_27	36.558	-98.89537	9	0.89/0.11/0	0/0.11/0.89	1/0/0	0/0.11/0.89
PET_28	36.79928	-98.24968	9	0.33/0.67/0	0.44/0.44/0.11	0.56/0.44/0	0/0/1
PET_29	36.9134	-99.23544	9	0.56/0.44/0	0.11/0.67/0.22	0.44/0.56/0	0/0/1
PET_30	36.40753	-99.55589	9	0.22/0.44/0.33	0.11/0.67/0.22	0.89/0.11/0	0/0/1
PET_31	35.19473	-100.72366	8	0.125/0.625/0.25	0.625/0.25/0.125	0.375/0.625/0	0/0/1
PET_32	31.74298	-110.68913	10	0/0/1	1/0/0	0/0/1	0/0/1
PET_33	31.47593	-110.25758	10	0/0/1	1/0/0	0/0/1	0/0/1
PET_34	31.38092	-110.06893	10	0/0/1	1/0/0	0/0/1	0/0/1
PET_35	31.68887	-109.1337	10	0/0/1	1/0/0	0/0/1	0/0/1
PET_43	32.42618	-106.54787	10	0/0/1	1/0/0	0/0/1	0/0/1
PET_46	31.43995	-102.87508	10	0/0/1	0.7/0.3/0	0/0.2/0.8	0/0/1
PET_47	31.63659	-102.81704	10	0.1/0.4/0.5	0/0/1	1/0/0	1/0/0
PET_48	31.71853	-103.03201	7	0/0/1	0.71/0.29/0	0.14/0.86/0	0/0/1
PET_49	31.95354	-102.97878	9	0.11/0.44/0.44	0/0.33/0.67	0.89/0.11/0	0.89/0.11/0
PET_50	32.50941	-102.04615	10	0/0.1/0.9	0.9/0.1/0	0/0.5/0.5	0/0/1

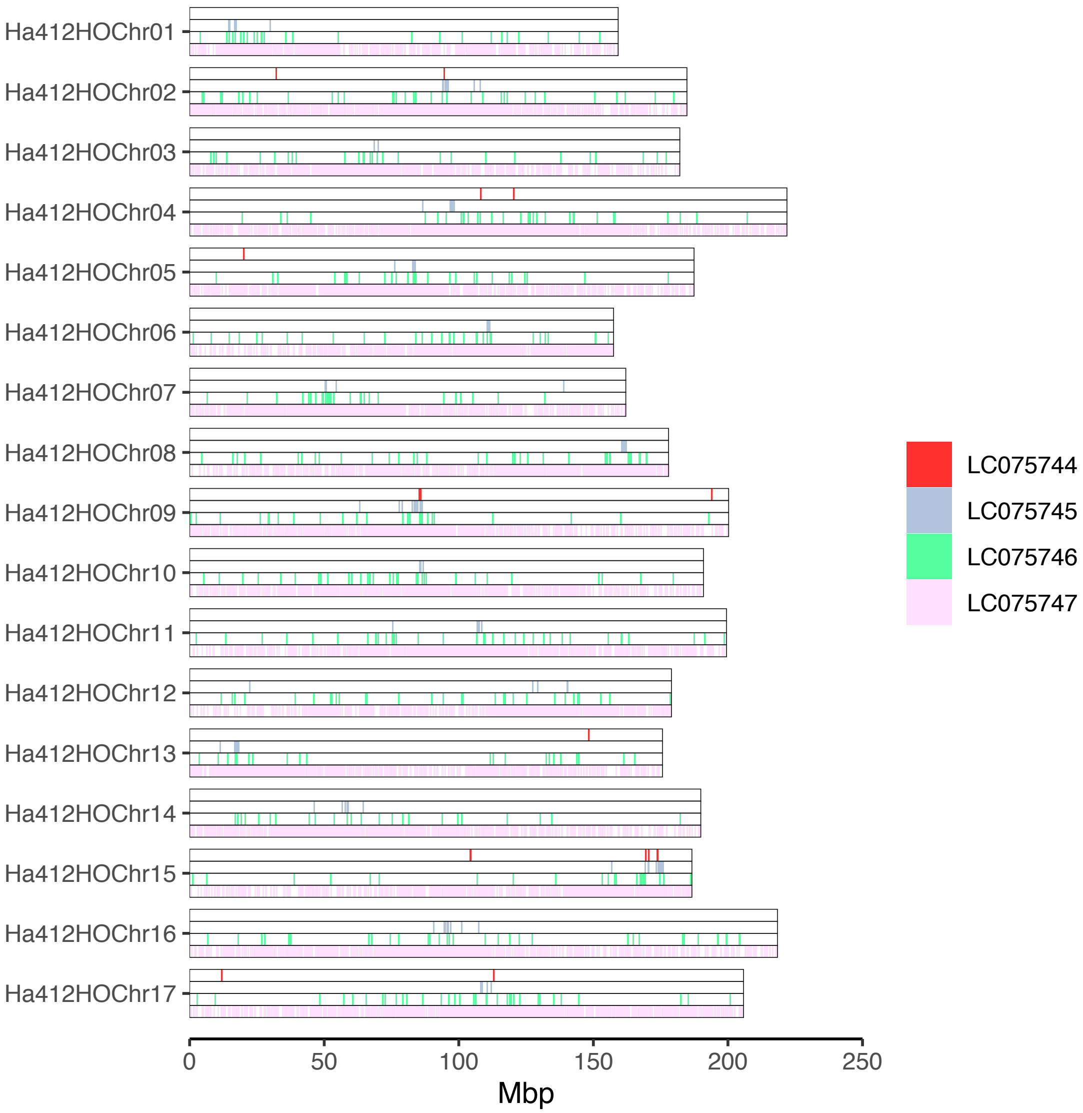

Figure S1. Distribution of the sequences previously found to be targeted by sunflower centromere-specific histone H3 across the 17 chromosomes of Ha412HOv2.0.

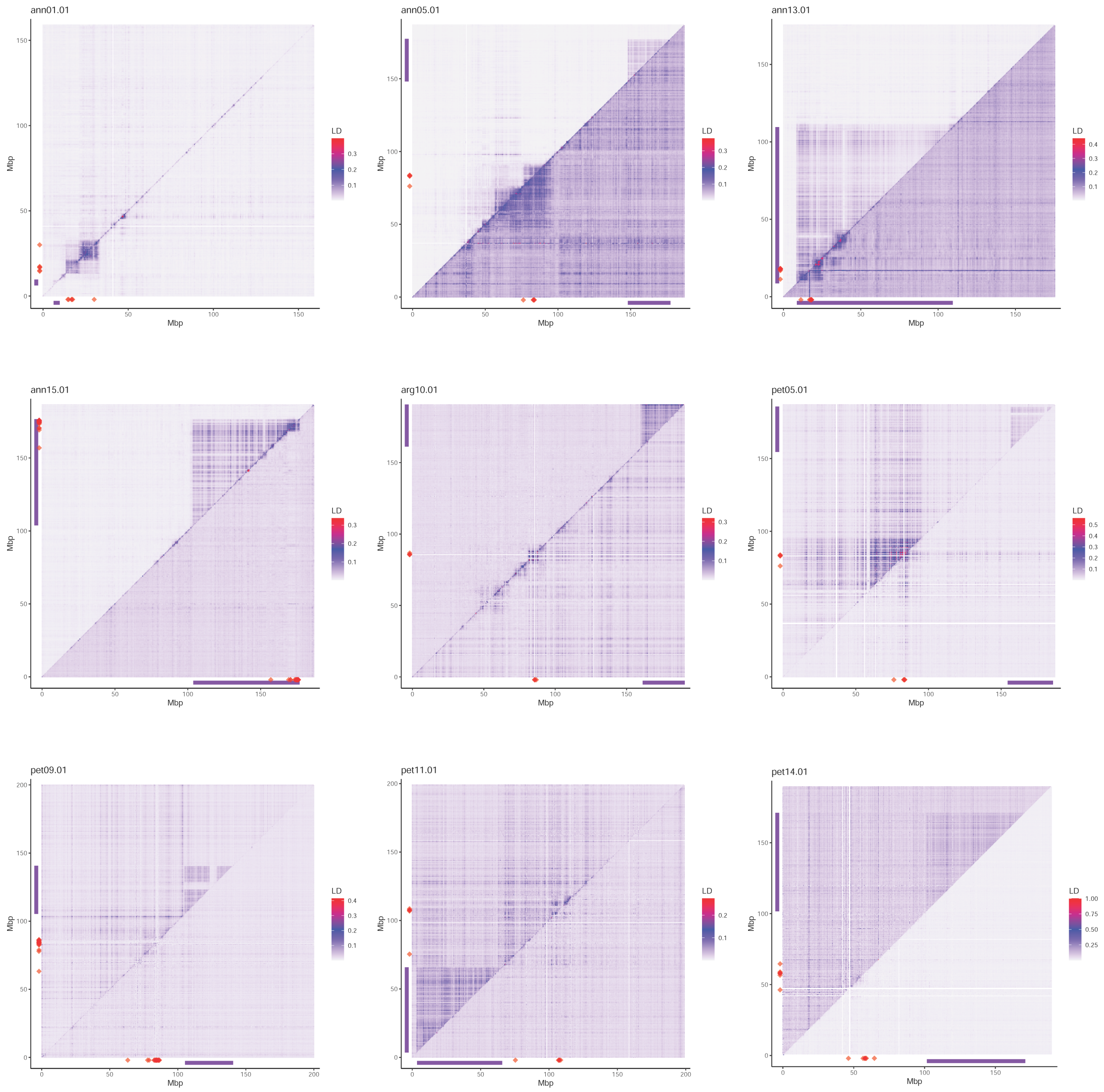

Figure S2. LD plots for all inversions. Upper triangle was based on all individuals from populations polymorphic for the inversion and lower triangle was based on individuals from populations homozygous for the more common orientation. Pairwise R^2 between SNPs were summarized and the average R^2 values were presented in 500-kbp windows. Purple bars represent the location of the inversion. Positions of the centromere-specific sequence LC075745

are indicated by red diamonds. Names for the inversions are consistent with those in Todesco et al. (2020).

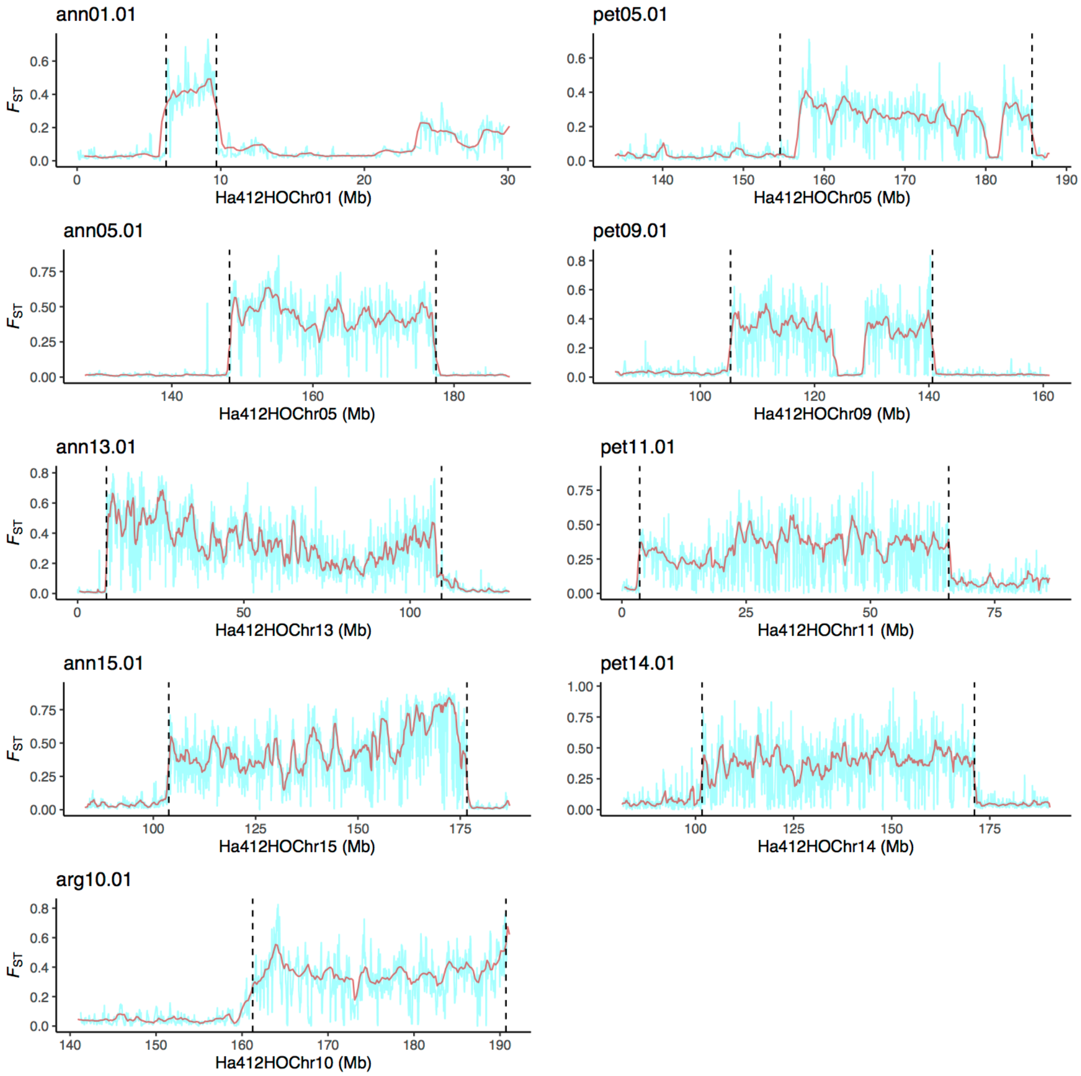

Fig. S3. Divergence (F_{ST}) between inversion orientations. Mean divergence is calculated over 1-Mbp sliding windows with a step size of 200-kbp (brown lines) and 100-kbp sliding windows with a step size of 20 kbp (cyan lines). Vertical dashed lines indicate the putative boundaries of each inversion.

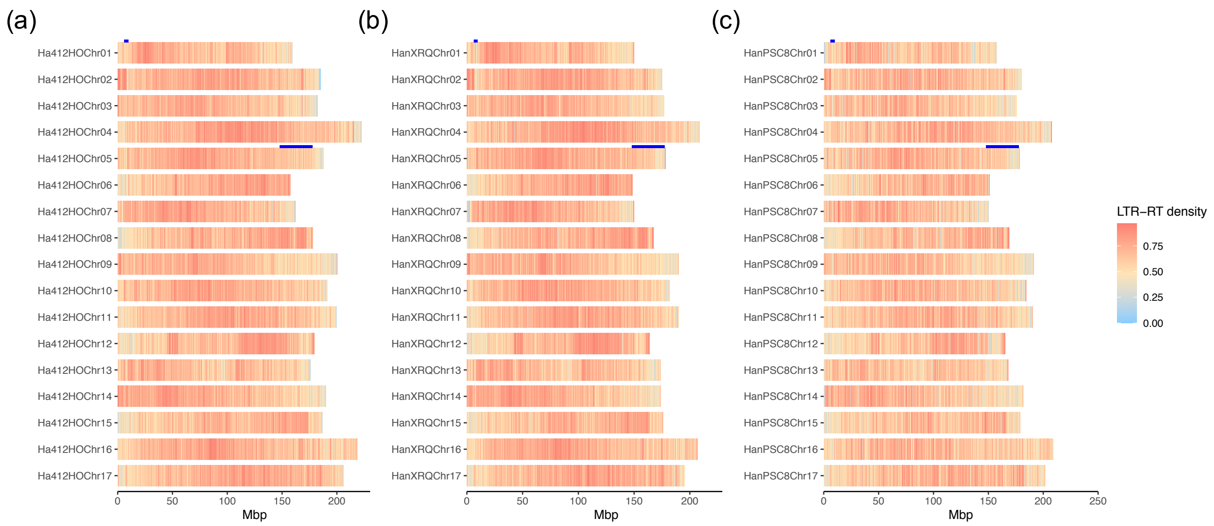

Figure S4. Genomic distributions of long terminal repeat retrotransposons (LTR-RTs) in the three genome assemblies of sunflower. (a) Ha412HOv2.0, (b) XRQv2, (c) PSC8. Density of LTR-RTs was calculated in 500 kbp bins per chromosome. Blue bars indicate the locations of inversions ann01.01 and ann05.01.

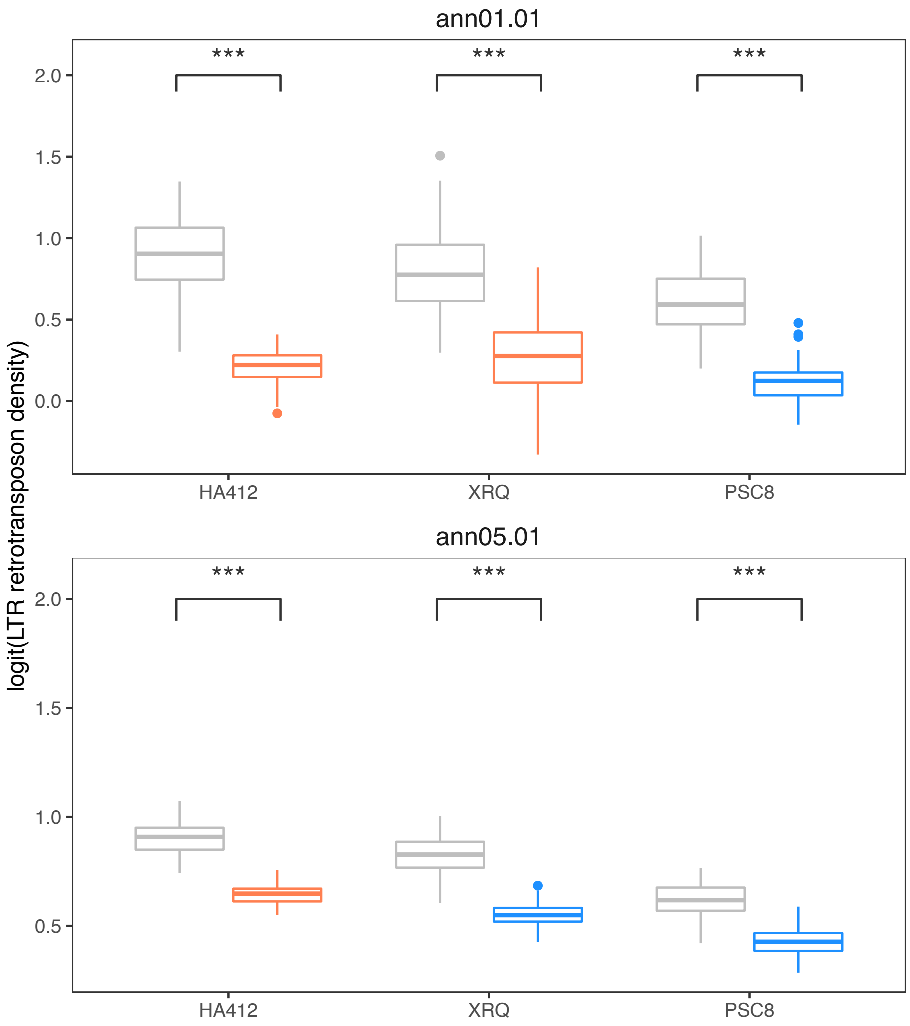

Figure S5. Long terminal repeat (LTR) retrotransposon density, computed on 500kb windows genome-wide (grey) as well as in forward (coral) and reverse (blue) arrangements of inversions ann01.01 (upper panel) and ann05.01 (lower panel) in Ha412HOv2, XRQv2 and PSC8 reference genomes. Asterisks denote significance in independent t-test: ***p<0.001.

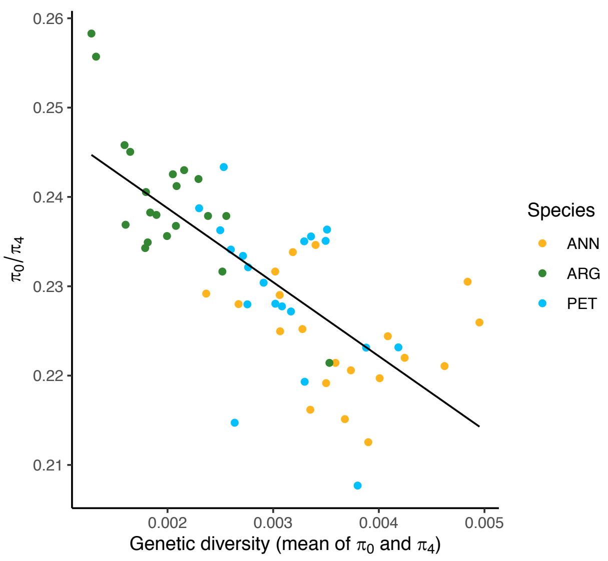

Figure S6. Genetic load (π_0/π_4) versus genetic diversity (mean of π_0 and π_4) across three sunflower species. The statistics were estimated with 20 individuals randomly chosen from each species of *Helianthus annuus* (ANN), *H. argophyllus* (ARG) and *H. petiolaris* (PET), respectively. The black line denotes the best-fit linear regression line. Both π_0 and π_4 were scaled by dividing by the standard deviation of each before the significance of the negative correlation between the variables represented by both axes was tested.

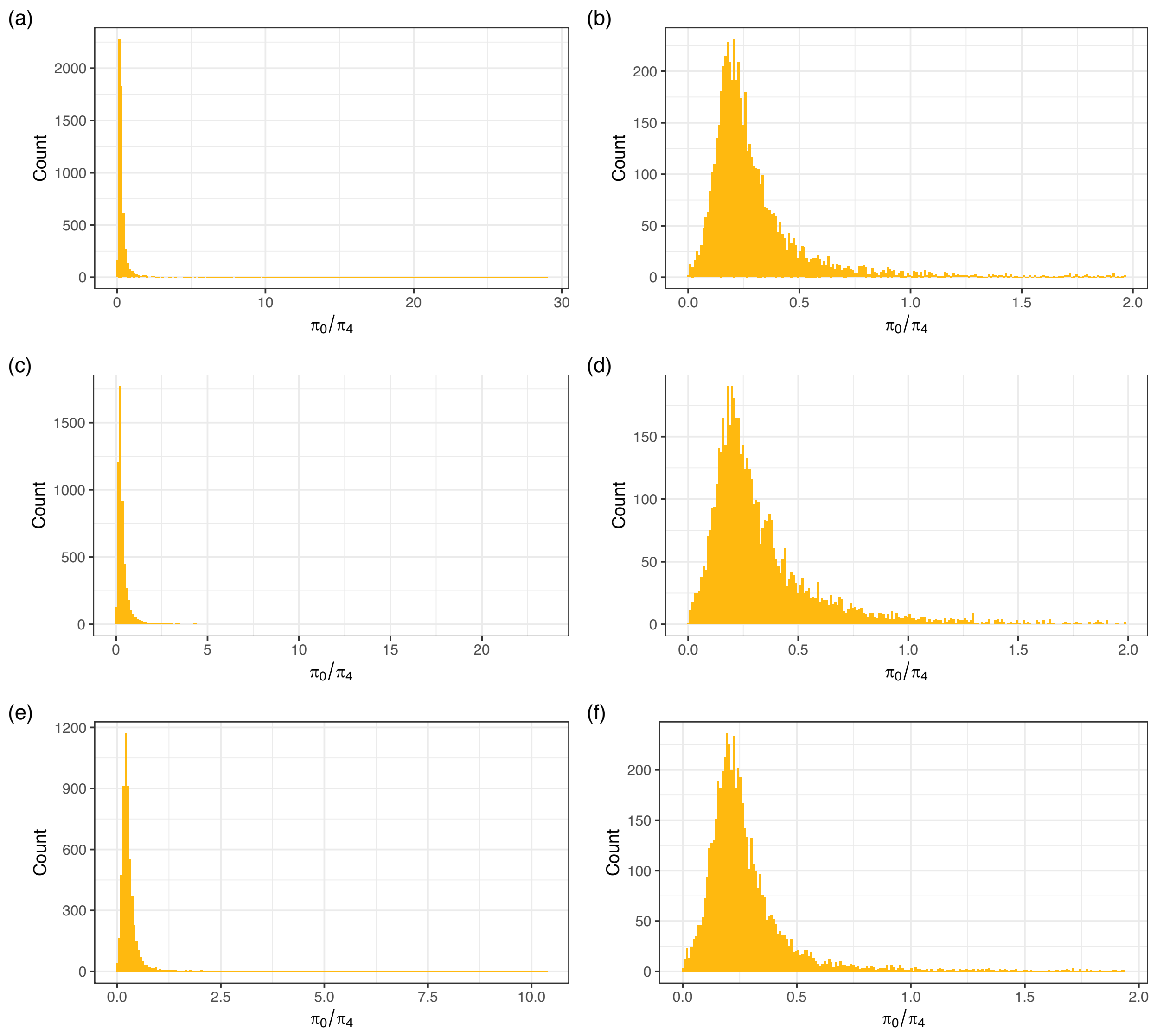

Figure S7. Distribution of window-based π_0/π_4 in each species. (a-b) *Helianthus annuus*, (c-d) *H. argophyllus*, (e-f) *H. petiolaris*. The ratio of π_0 and π_4 was calculated in sliding windows of 500 kbp. (a), (b) and (c) are distributions for all windows across the genomes**;** (b), (d) and (f) are distributions for windows with values < 2.

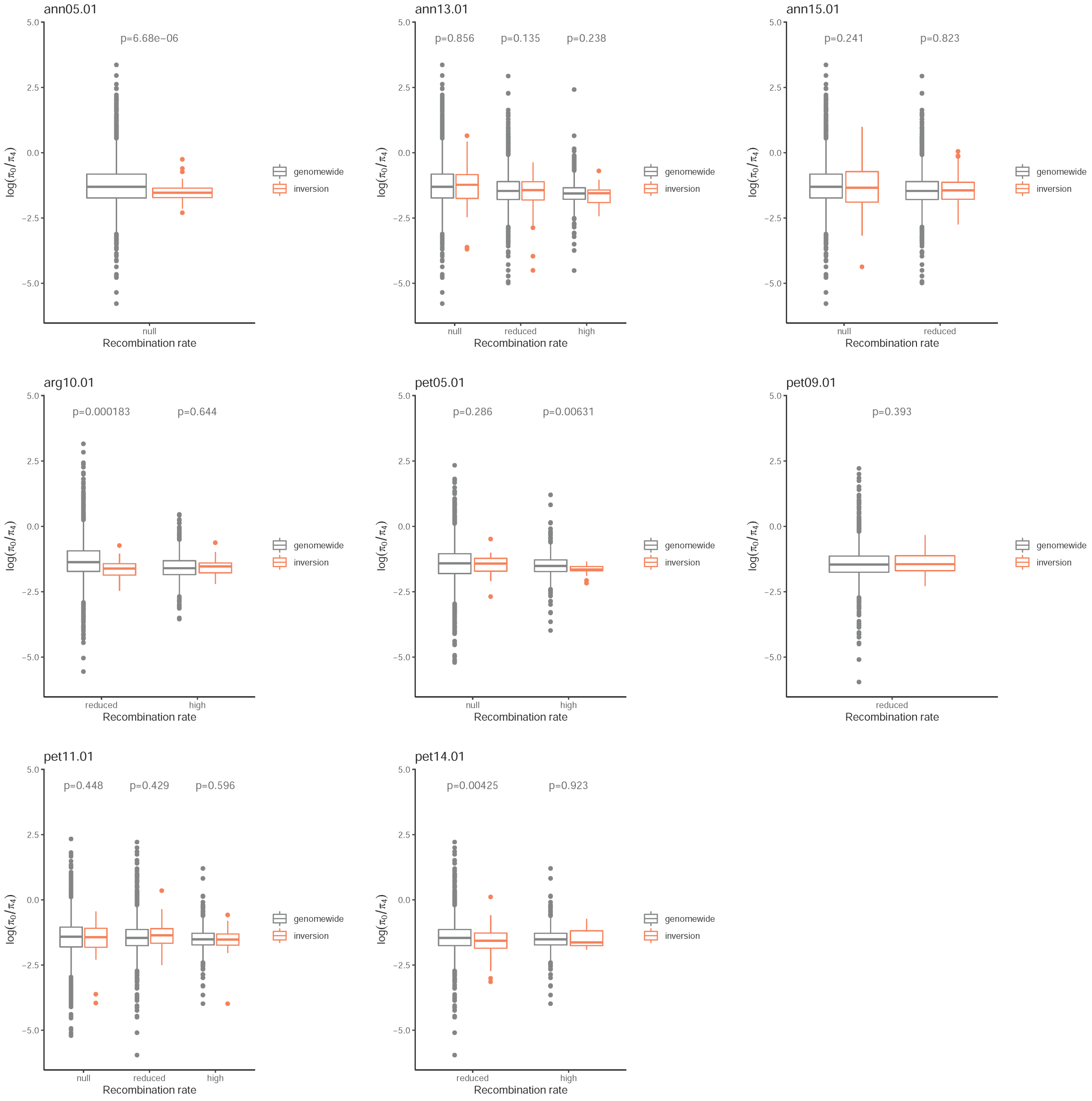

Figure S8. Comparison of π_0/π_4 within inversions to genome-wide regions in the same recombination rate category using all samples. Windows of each recombination rate category were compared separately. P-values are Welch two sample t-test on log-transformed π_0/π_4 values.

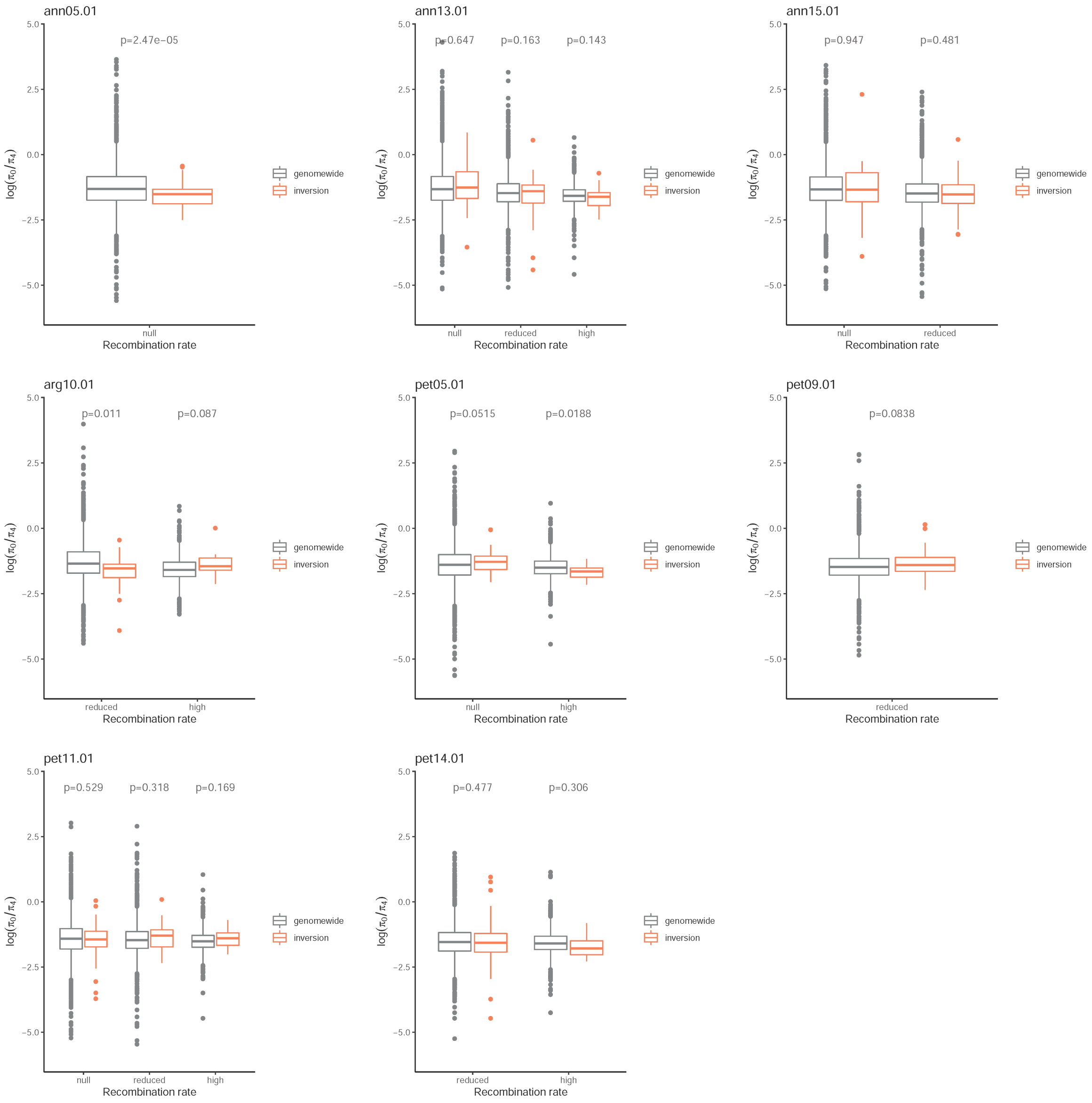

Figure S9. Comparison of π_0/π_4 within inversions to genome-wide regions in the same recombination rate category using samples homozygous for the rarer arrangement. Windows of each recombination rate category were compared separately. P-values are Welch two sample t-test on log-transformed π_0/π_4 values.

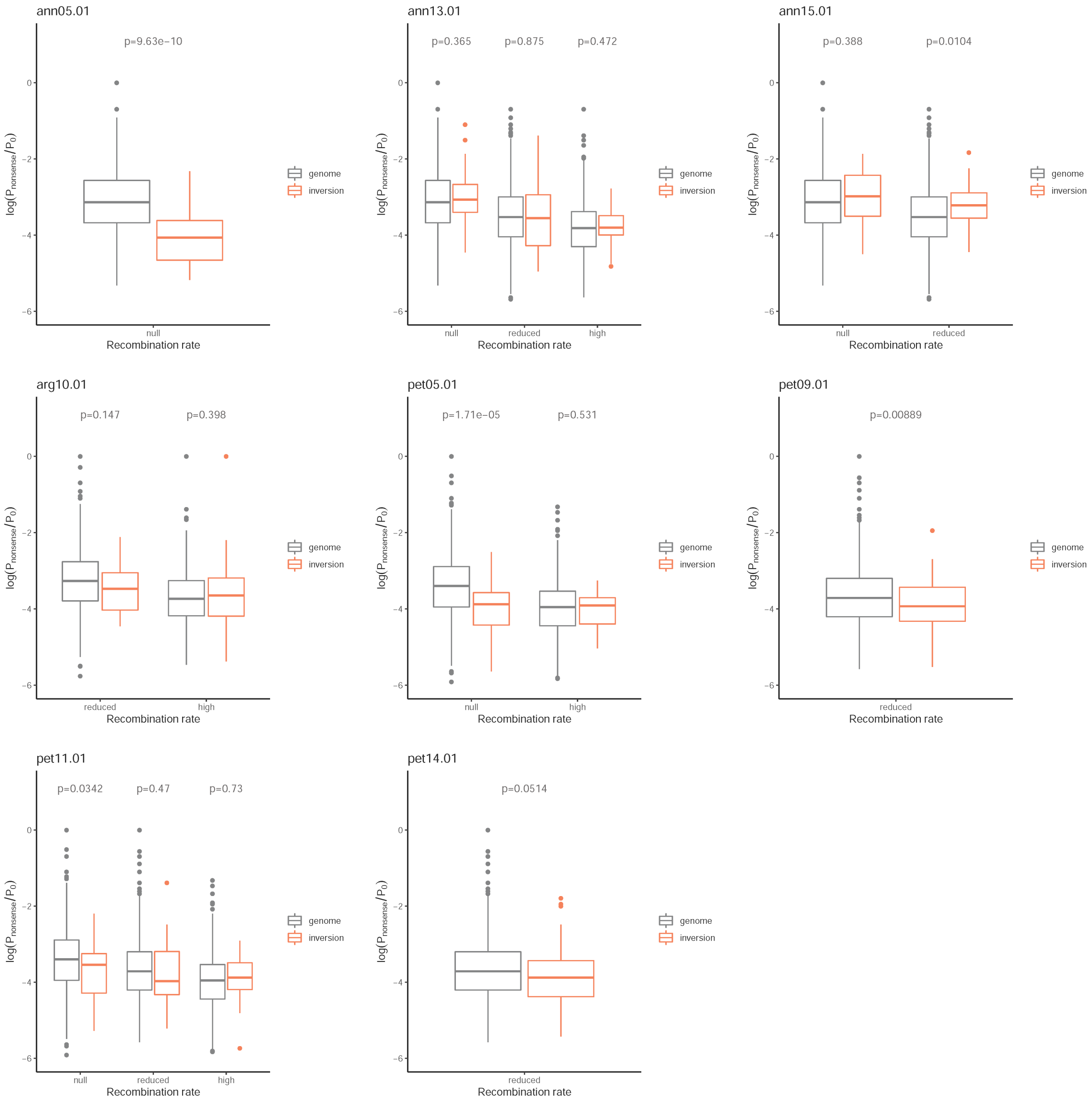

Figure S10. Comparison of $P_{nonsense}/P_0$ within inversions to genome-wide regions in the same recombination rate category using all samples. Windows of each recombination rate category were compared separately. P-values are Welch two sample t-test on log-transformed $P_{nonsense}/P_0$ values.

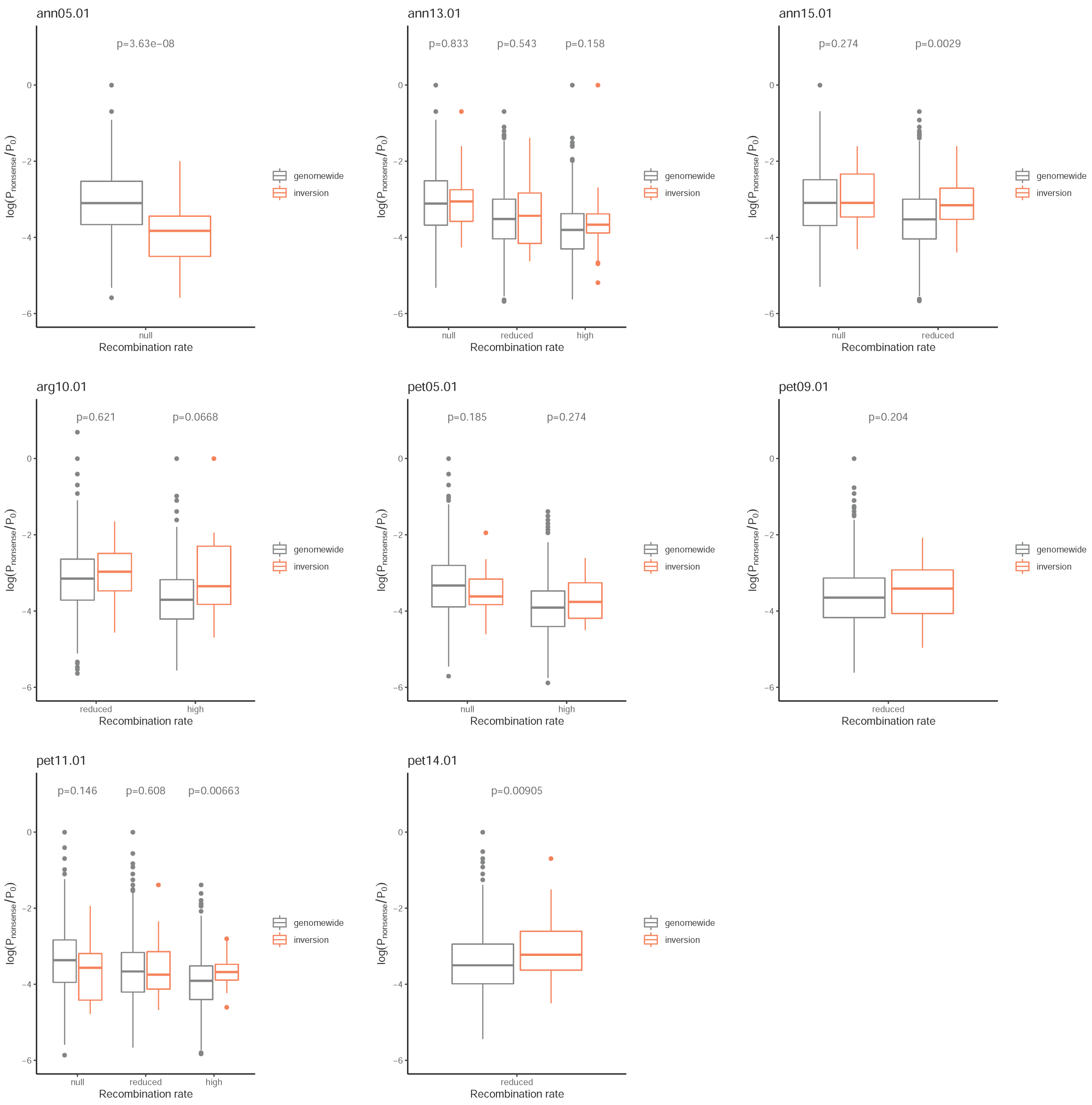

Figure S11. Comparison of $P_{\mathrm{nonsense}}/P_0$ within inversions to genome-wide regions in the same recombination rate category using samples homozygous for the rarer arrangement. Windows of each recombination rate category were compared separately. P-values are Welch two sample t-test on log-transformed $P_{\mathrm{nonsense}}/P_0$ values.

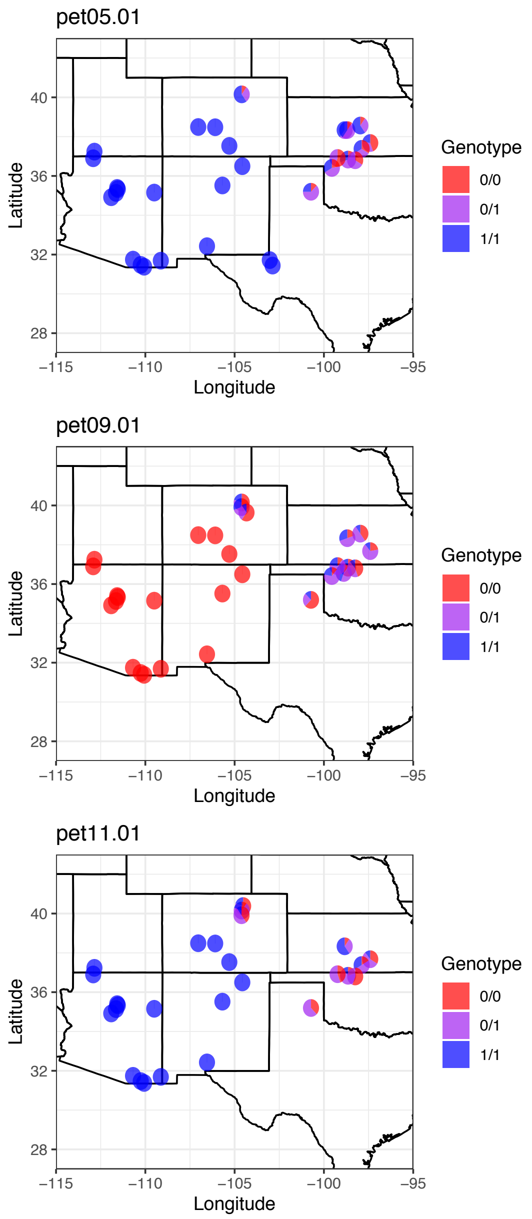

Figure S12. Distribution maps of inversion genotypes in populations used in the comparison of deleterious mutations between monomorphic populations and polymorphic populations for inversion pet05.01, pet09.01, and pet11.01. Monomorphic populations contain one arrangement for the inversion (genotype 0/0 or 1/1), and polymorphic populations contain multiple genotypes for the inversions.

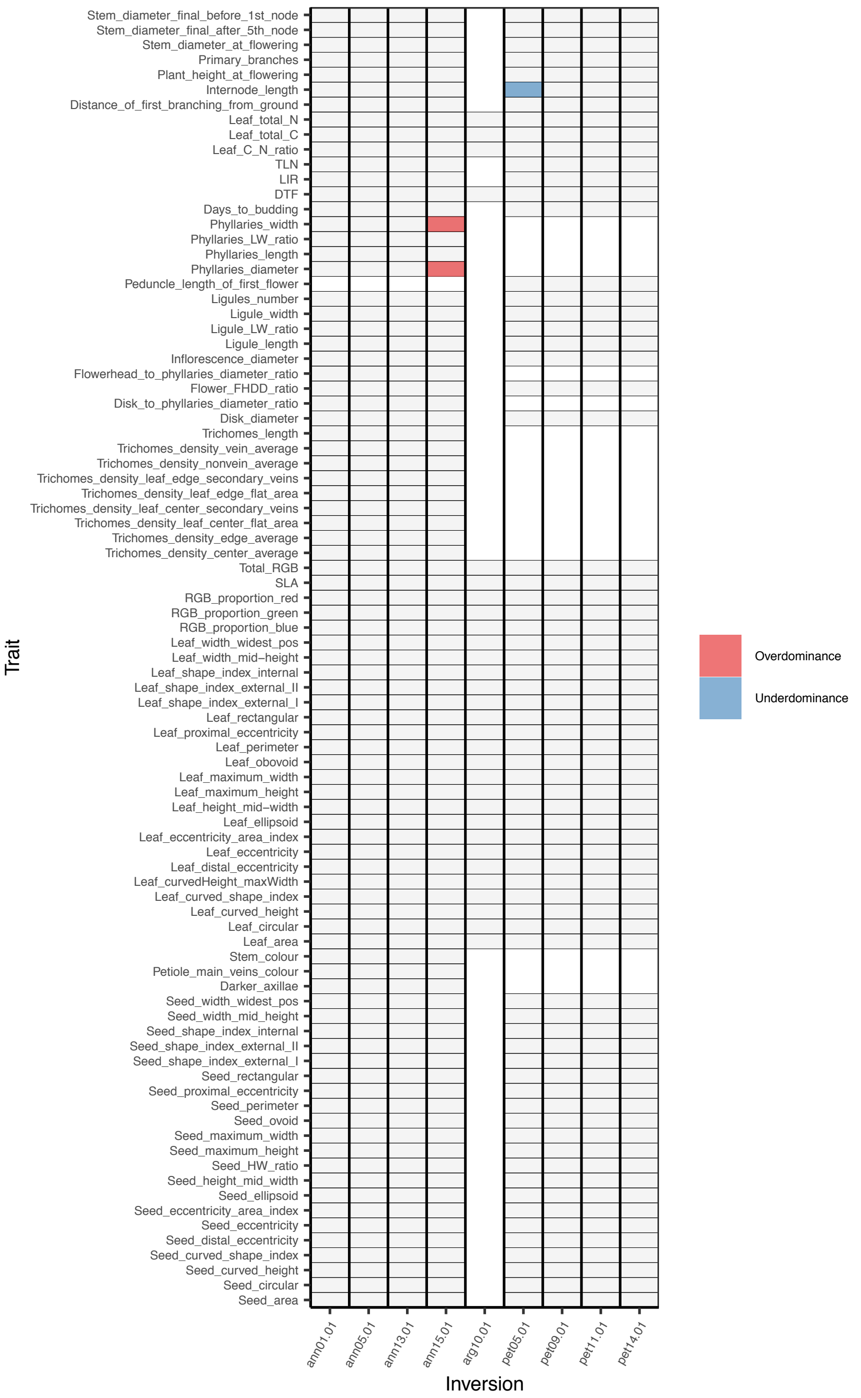

Figure S13. Survey of phenotypic traits across inversion genotypes. Traits surveyed for each inversion are labeled in grey. The cases where heterozygotes have a significantly (p < 0.01)

greater or lower phenotype than both homozygotes are shaped in color according to their condition. Trait names and details are described in Todesco et al. (2020).

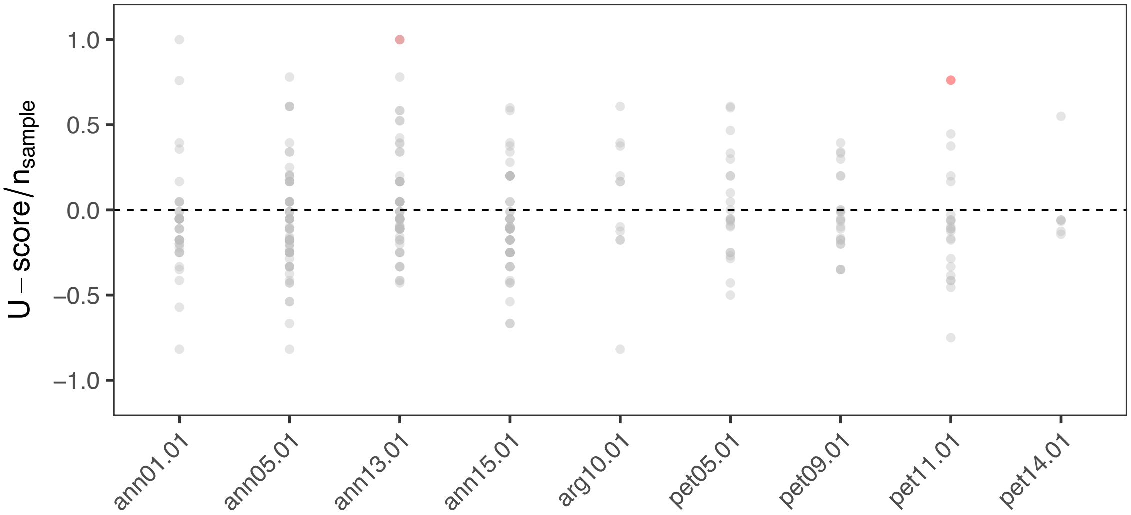

Figure S14. Population U-scores for inversions. U-scores were calculated for each population for each inversion and scaled by number of samples. A positive U-score indicates excess of homozygotes while a negative value indicates overrepresentation of heterozygotes. Values significantly ($p<0.01$) different from zero are marked in red.
